# Association Between Malocclusion Severity and Oral Health‐Related Quality of Life in Children Aged 11–14 Years Using CPQ11‐14 and P‐CPQ

**DOI:** 10.1002/cre2.70337

**Published:** 2026-03-26

**Authors:** Seyedeh Hediyeh Daneshvar, Negin Bazgir, Sobhan Agheshteh Sabzevar, Yasamin Babaee Hemmati

**Affiliations:** ^1^ Department of Pediatric Dentistry, Dental Sciences Research Center, School of Dentistry Guilan University of Medical Sciences Rasht Iran; ^2^ School of Dentistry Guilan University of Medical Sciences Rasht Iran; ^3^ Student Research Committee, School of Dentistry Guilan University of Medical Sciences Rasht Iran; ^4^ Department of Orthodontics, Dental Sciences Research Center, School of Dentistry Guilan University of Medical Sciences Rasht Iran

**Keywords:** child oral health, cross‐sectional studies, malocclusion, oral health‐related quality of life

## Abstract

**Objectives:**

This study aimed to evaluate the association between malocclusion severity and oral health‐related quality of life (OHRQoL) in children aged 11–14 years by simultaneously using the Child Perceptions Questionnaire (CPQ11‐14), Parent/Caregiver Perceptions Questionnaire (P‐CPQ), and Dental Aesthetic Index (DAI).

**Material and Methods:**

This analytical cross‐sectional, clinic‐based study was conducted on 117 children aged 11–14 years and their parents/guardians referred to a specialized dental clinic in Rasht city, Iran, in 2022. Children with systemic diseases, a history of orthodontic treatment, or craniofacial abnormalities were excluded. OHRQoL was independently assessed using the CPQ11‐14 and P‐CPQ. Malocclusion severity was determined using the DAI, and children were categorized into normal occlusion, definite, severe, and very severe malocclusion groups. The associations of age and gender with OHRQoL were also examined. Data were analyzed statistically (*α* = 0.05).

**Results:**

The mean OHRQoL score was significantly higher among children with malocclusion compared with those with normal occlusion (*p* < 0.001), indicating poorer quality of life. Higher malocclusion severity was significantly associated with worse OHRQoL scores (*p* < 0.001). These associations were significant in both sexes (*p* < 0.05). A significant association between malocclusion severity and poorer OHRQoL was observed in children older than 12 years (*p* < 0.001), whereas this association was not statistically significant in children aged 12 years or younger (*p* > 0.05). Multivariable analysis confirmed that malocclusion severity remained independently associated with OHRQoL after adjustment for demographic factors (*p* < 0.001).

**Conclusions:**

Greater severity of malocclusion, particularly in older children, is significantly associated with poorer OHRQoL. The relative agreement between child and parent assessments highlights the importance of considering both perspectives in clinical and research settings.

## 1 Introduction

Malocclusion is a developmental condition in which there is a deviation from the normal relationship of the teeth with each other in the same arch or in the opposite arch, which can cause deformity or dysfunction (Londono et al. 2023). Malocclusion is primarily the result of tooth‐size arch‐size discrepancy, which is largely determined genetically (J. Baskaradoss and Bhagavatula 2021). However, the etiology of malocclusion is often multifactorial, and less than 5% of the population may have a pattern of malocclusion that can be attributed to a specific cause. Other causes include fetal growth and developmental defects, trauma, postnatal developmental abnormalities, and environmental factors (hormonal abnormalities, temporomandibular joint disorders, and bad habits such as thumb/lip sucking) (Kenza et al. 2025; Rai et al. 2022).

Malocclusion is the third most common oral disease after dental caries and periodontal disease, and about half of children and adolescents worldwide suffer from some form of it (Atasever İşler et al. 2025). Its prevalence is 54% in the primary dentition period, which remains constant during the permanent dentition period (Lombardo et al. 2020). In general, there is no significant difference between girls and boys in terms of incidence of malocclusion. However, tooth eruption and maturation usually occur faster in girls than in boys, and girls are usually affected by malocclusion earlier than boys (Lagorsse and Gebeile‐Chauty 2018). Ethnic differences, regional economic differences, dietary habits, geographical location, and time of study cause variations in the prevalence and type of malocclusion in different populations reported in the literature (Yin et al. 2023). According to a systematic review conducted in 2018, the overall prevalence of malocclusion in the Iranian population was about 87% on average at the time. However, the prevalence of malocclusion varied considerably across studies (ranging from 23.7% to 99.7%) (Eslamipour et al. 2018).

There are no specific clinical guidelines for determining the need for orthodontic treatment or the timing of orthodontic interventions; however, several attempts have been made to develop standardized quantitative indices to assess the orthodontic treatment need that can be applicable in both clinical settings and epidemiological studies (Nugroho et al. 2019). Internationally recognized indices include the Index of Orthodontic Treatment Need, the Index of Complexity, Outcome, and Need, and the Dental Aesthetic Index (DAI) (Angkana et al. 2021). The DAI was first introduced by Cons et al. (1989). This index is based on the patient's esthetic, psychological, and social factors. It evaluates 10 features that are multiplied by their respective weights and summed for the final DAI score. Finally, the patients are classified into four levels of malocclusion (Cons et al. 1989).

The widespread consequences of malocclusion include impaired mastication and deglutition function, speech problems, temporomandibular joint disorders, and bruxism, negatively impacting the esthetic appearance, mental health, and oral health‐related quality of life (OHRQoL) of patients (Jafari et al. 2024; Vejdani et al. 2019). According to the World Health Organization, oral health is determined based on variables such as caries indices, periodontal parameters, general well‐being, and the ability to eat and speak (Glick et al. 2012). The assessment of OHRQoL reflects the interaction of an individual's oral health status with social and contextual factors, and should be considered as a component when assessing the oral health status (Jain et al. 2021). Several measures have been developed to assess the OHRQoL, the most common of which is the Child Perceptions Questionnaire (CPQ). The CPQ has been validated in different versions for different age groups, including CPQ8‐10, CPQ11‐14, CPQ14‐16, and CPQ 28‐100. The CPQ11‐14 consists of 37 questions, and assesses the consequences of oral health problems and their effects on the quality of life of children between 11 and 14 years of age (De Stefani et al. 2019; Gomasani et al. 2025).

Several studies have investigated the impact of malocclusion on OHRQoL of children and adolescents, often using standardized questionnaires such as the CPQ and the malocclusion severity assessment indices (Nosratabadi et al. 2023; J. K. Baskaradoss et al. 2022; Duisterwinkel et al. 2025; Noronha et al. 2025; Piassi et al. 2019; da Rosa et al. 2016; Rantavuori et al. 2023; Kassis et al. 2018). A 2019 study by Piassi et al. (2019) showed that malocclusion in the mixed dentition period affected the children's OHRQoL, but malocclusion severity did not have a significant impact on the quality of life. Also, Kavaliauskienė et al. (2019) validated the Lithuanian version of the CPQ11‐14 and showed that this tool was reliable for measuring the OHRQoL of 11–14‐year‐old children, and that children and parents' responses were significantly correlated. However, relatively small sample size (Duisterwinkel et al. 2025), not using valid and comprehensive indices to assess the malocclusion severity (Duisterwinkel et al. 2025), lack of simultaneous consideration of children and parents' perspectives (Noronha et al. 2025; Kassis et al. 2018), and limitations related to study design, especially the use of cross‐sectional design in studies (Noronha et al. 2025; Rantavuori et al. 2023), reduce the generalizability of the findings. While similar studies are present in Iran, important methodological differences highlight the importance of the present study (Heravi et al. 2011; Dalaie et al. 2018; Sobouti, Armin, et al. 2020; Sobouti, Kavianpour, et al. 2020; Khanemasjedi et al. 2018).

Some of the available studies assessed the quality of life in general, and did not specifically focus on OHRQoL (Sobouti, Armin et al. 2020; Sobouti, Kavianpour, et al. 2020). They typically used indices such as the Oral Health Impact Profile and the Orthognathic Quality of Life Questionnaire, (Dalaie et al. 2018; Sobouti, Armin et al. 2020; Sobouti, Kavianpour, et al. 2020) and also focused on adult or adolescent populations outside the 11–14‐year age range (Heravi et al. 2011; Dalaie et al. 2018; Sobouti, Armin et al. 2020; Sobouti, Kavianpour, et al. 2020; Khanemasjedi et al. 2018). In addition, they used different orthodontic indices such as the Index of Orthodontic Treatment Need (Dalaie et al. 2018; Sobouti, Armin et al. 2020; Sobouti, Kavianpour, et al. 2020; Khanemasjedi et al. 2018) and the Index of Complexity, Outcome, and Need (Heravi et al. 2011), while the DAI has not been consistently addressed. The present study attempted to assess the relationship between the malocclusion severity and OHRQoL more accurately and comprehensively by utilizing a relatively larger sample size (compared to previous studies), using the validated CPQ11‐14 and P‐CPQ, and assessing the severity of malocclusion with the DAI, while simultaneously considering the perspectives of children and parents. Therefore, the aim of the present study was to evaluate the association between malocclusion severity and OHRQoL in children aged 11–14 years using the CPQ11‐14 and P‐CPQ.

## 2 Materials and Methods

This analytical cross‐sectional study was conducted to evaluate the association between malocclusion severity and OHRQoL in children aged 11–14 years. The research protocol was approved by the Ethics Committee of Guilan University of Medical Sciences (Ethics code: IR.GUMS.REC.1400.616). Written informed consent was obtained from parents or guardians prior to participation.

Convenience sampling was used to recruit eligible participants. Children aged 11–14 years who presented to a specialized dental clinic in Rasht city, Iran, in 2022, and met the inclusion criteria were enrolled until the required sample size was achieved. This sampling approach was selected due to accessibility to the target population and the feasibility of accurate eligibility assessment. A total of 117 children and their parents/guardians were included in the study.

OHRQoL was assessed independently from both the child's and the parent's perspectives using the CPQ11‐14 and P‐CPQ questionnaires, respectively. To prevent mutual influence, children and parents completed the questionnaires separately and were instructed not to communicate during completion. Any questions were addressed by the researcher. Following questionnaire completion, a clinical oral examination was performed by an experienced orthodontist.

The CPQ11‐14 and P‐CPQ questionnaires were translated into Persian using a standard forward–backward translation procedure. Two independent bilingual dental professionals performed the forward translation. Backward translation was conducted by an independent bilingual translator who was blinded to the original versions. The pre‐final Persian versions were reviewed by a panel of five dental and orthodontic experts to ensure conceptual equivalence, clarity, and cultural appropriateness.

A pilot test was conducted on 20 children and their parents to evaluate item clarity and comprehensibility. Feedback from pilot testing indicated that no major cultural modifications to the questionnaire items were required.

Sample size estimation was based on the formula for comparing mean CPQ total scores between groups, assuming a statistical power of 80%, a significance level of 0.05, standard deviations of 6.2 and 5.6, and a mean difference of 3.5 units. The minimum required sample size was calculated as 45 participants per group, with at least 15 participants considered for each level of malocclusion severity. To ensure adequate representation across severity categories, additional participants were recruited, resulting in a final sample of 117 children.

The inclusion criteria included age 11–14 years, presence of at least one parent/guardian, complete eruption of permanent anterior teeth and first molars, children and parents/guardians perfectly capable of cooperating in clinical examination and completing the questionnaires, and children with good general health status with no history of orthodontic treatment and not being in an active phase of orthodontic treatment.

The exclusion criteria were systemic diseases such as diabetes mellitus and metabolic diseases, cognitive disorders or mental disorders that would prevent accurate performance of tests and self‐report sections, and children with congenital craniofacial anomalies, trauma to the orofacial region, or severe dental anomalies that could affect the results of malocclusion or quality of life assessments. The presence of extensive untreated caries or missing teeth and lack of cooperation in clinical examination or completing the questionnaire were also considered among the exclusion criteria (Piassi et al. 2019).

Malocclusion severity was assessed using the DAI, which comprises 10 occlusal characteristics, including anterior tooth loss, incisal crowding and spacing, midline diastema, maxillary and mandibular anterior crowding, maxillary and mandibular overjet, anterior open bite, and molar anteroposterior relationship. All DAI assessments were performed by a single calibrated orthodontist using a dental mirror and a millimeter‐scale periodontal probe under standardized lighting and seating conditions. The examiner was trained according to the official DAI protocol prior to data collection. Duplicate measurements were obtained for a random subset of 15 children within a 2‐week interval to evaluate intra‐examiner reliability. Excellent reproducibility was observed (ICC = 0.98; *r* = 0.87), confirming high measurement consistency. Based on DAI scores, participants were categorized as follows (Chandra 2023):

DAI ≤ 25: Normal occlusion or mild malocclusion.
DAI between 26 and 30: Definite malocclusion.
DAI between 31 and 35: Severe malocclusion.
DAI ≥ 36: Very severe malocclusion.

Children with DAI scores ≤ 25 were considered to have normal or mild malocclusion, whereas those with DAI scores ≥ 26 were classified into increasing levels of malocclusion severity.

Data on children's OHRQoL were collected using the CPQ11‐14. This questionnaire was given to the child and completed by him/her after a brief explanation. The scores of the questions were tabulated according to the numerical codes intended for the answers, such that 4 was for “every day/almost every day,” 3 for “often,” 2 for “sometimes,” 1 for “once or twice,” and zero was for “never.” The CPQ11‐14 consists of 37 questions distributed in four health domains, including oral symptoms (6 questions), functional limitations (9 questions), emotional health (9 questions), and social health (13 questions). The total score of the CPQ11‐14 ranges from 0 to 148. A higher score indicates a lower quality of life (De Stefani et al. 2019).

Data on parents' perceptions of their children's OHRQoL were collected using the P‐CPQ. This questionnaire was administered to, and completed by each child's parent/guardian. Demographic information on age, gender, parental education, and family income was collected with questions at the beginning of the questionnaire. The scores of the questions were tabulated according to the numerical codes assigned to the responses, such that 4 was for “every day/almost every day,” 3 for “often,” 2 for “sometimes,” 1 for “once or twice,” and 0 for “never.” This instrument consists of 35 items across four domains: oral symptoms (6 items), functional limitations (8 items), emotional well‐being (9 items), and social well‐being (12 items). In addition, two global questions assess parents' perceptions of their child's overall oral health and the overall impact of oral conditions on the child's life. The total score of the P‐CPQ ranges from 0 to 140. A higher score indicates a lower quality of life (Çınar et al. 2025).

In this study, the CPQ11‐14 and P‐CPQ were used based on the standard forward‐backward process and cultural adaptation. The content validity of the CPQ11‐14 and P‐CPQ was confirmed by the opinion of five dental and orthodontic experts, and the content validity ratio and content validity index were calculated to be 0.99 and 0.98, respectively, indicating adequate validity. To measure reliability, the Cronbach's alpha coefficient was calculated to be 0.86 for CPQ11‐14 and 0.83 for P‐CPQ, indicating the appropriate reliability of these questionnaires. These results are in accordance with international validation studies of the aforementioned instruments and provide assurance of the quality of the collected data (Jokovic et al. 2002).

### 2.1 Statistical Analysis of the Data

Statistical analysis was performed using SPSS software version 26 (IBM Corp., Armonk, NY, USA). Data distribution was assessed using the Shapiro–Wilk test, along with skewness and kurtosis indices. Homogeneity of variances across groups was evaluated using Levene's test. For quantitative variables, when assumptions of normality and homogeneity of variances were met, independent *t*‐tests were used for two‐group comparisons, and one‐way analysis of variance (ANOVA) was applied for comparisons across more than two groups. When ANOVA results were statistically significant, post hoc pairwise comparisons were conducted using Tukey's test or Games–Howell test, as appropriate. If the assumptions for parametric tests were not satisfied, non‐parametric alternatives including the Mann–Whitney *U* test and Kruskal–Wallis test were employed. Effect sizes were calculated to quantify the magnitude of group differences. For ANOVA models, eta squared (*η*²) was reported, and for Kruskal–Wallis tests, epsilon squared (*ε*²) was calculated. For pairwise comparisons, standardized mean differences (Cohen's *d*) were computed. In addition, multivariable linear regression analyses were performed to evaluate the independent association between malocclusion severity and OHRQoL outcomes. Separate models were constructed for CPQ11‐14 and P‐CPQ scores. The DAI was entered as the main independent variable, and the models were adjusted for child age, child sex, parental education level, and family income. Model assumptions, including normality of residuals, homoscedasticity, and multicollinearity, were assessed and found to be acceptable. The level of statistical significance was set at *p* < 0.05 for all tests.

## 3 Results

A total of 117 children aged 11–14 years were included in the study. Based on the DAI, 66 children (56.4%) had normal occlusion or mild malocclusion (DAI ≤ 25), while 51 children (43.6%) had malocclusion (DAI ≥ 26). Among children with malocclusion, 19 (16.2%) had definite malocclusion, 15 (12.8%) had severe malocclusion, and 17 (14.5%) had very severe malocclusion. The mean DAI score of the study population was 21.99 ± 12.67. The overall mean oral health‐related quality of life (OHRQoL) score was 38.98 ± 24.83 based on the CPQ11‐14 questionnaire and 37.14 ± 23.03 based on the P‐CPQ questionnaire.

Children with malocclusion had significantly poorer OHRQoL compared with those with normal occlusion or mild malocclusion. The mean CPQ11‐14 score was significantly higher in the malocclusion group than in the DAI ≤ 25 group (51.90 ± 26.97 vs. 29.00 ± 17.56; *p* < 0.001). Similarly, P‐CPQ scores were significantly higher among children with malocclusion compared with the DAI ≤ 25 group (48.96 ± 26.14 vs. 28.00 ± 15.40; *p* < 0.001) (Table 1). These differences were not only statistically significant but also clinically meaningful, as reflected by large effect sizes and corresponding 95% confidence intervals.

**Table 1:** Comparison of CPQ11‐14 and P‐CPQ scores between children with malocclusion (DAI ≥ 26) and those with normal/mild malocclusion (DAI ≤ 25).

	Patient	CPQ11‐14	P‐CPQ	Test statistics^a^		*p* value	
Group	*n* (%)	(mean ± SD)	(mean ± SD)	CPQ11‐14	P‐CPQ	CPQ11‐14	P‐CPQ
DAI ≥ 26 group	51 (43.6%)	51.90 ± 26.97	48.96 ± 26.14	5.26	−5.10	< 0.001	< 0.001
DAI ≤ 25 group	66 (56.4%)	29.00 ± 17.56	28.00 ± 15.40				

*Note:* In addition to statistical significance, the magnitude of the differences was clinically relevant. For CPQ11‐14, the mean difference between children with malocclusion (DAI ≥ 26) and those with normal or mild malocclusion (DAI ≤ 25) was 22.9 points (95% CI: 15.2–30.6), corresponding to a large effect size (Cohen's *d* = 1.04). Similarly, for P‐CPQ, the mean difference was 21.0 points (95% CI: 13.6–28.3), also indicating a large effect size (Cohen's *d* = 1.00).

Abbreviations: DAI, Dental Aesthetic Index; DAI ≤ 25, normal occlusion or mild malocclusion; DAI ≥ 26, definite, severe, or very severe malocclusion.

Higher CPQ11‐14 and P‐CPQ scores indicate poorer oral health‐related quality of life (OHRQoL).

^a^ Independent samples *t*‐test.

A significant increase in OHRQoL impairment was observed with increasing malocclusion severity. Mean CPQ11‐14 scores increased from 36.47 ± 12.64 in children with definite malocclusion to 46.07 ± 21.68 in those with severe malocclusion and 74.29 ± 28.90 in those with very severe malocclusion (*p* < 0.001). A similar trend was observed for P‐CPQ scores, with mean values of 32.78 ± 14.35, 45.53 ± 22.99, and 70.06 ± 25.45 for definite, severe, and very severe malocclusion, respectively (*p* < 0.001) (Table 2). Post hoc pairwise comparisons demonstrated that children with very severe malocclusion had significantly higher CPQ11‐14 and P‐CPQ scores compared with those with definite malocclusion (*p* < 0.01). Differences between the definite and severe malocclusion groups were smaller and were not consistently statistically significant.

**Table 2:** CPQ11‐14 and P‐CPQ scores across malocclusion severity categories (DAI ≥ 26 only).

	Patient	CPQ11‐14	P‐CPQ	Test statistics		*p* value^a^ (effect size)	
Malocclusion	*n* (%)	(mean ± SD)	(mean ± SD)	CPQ11‐14	P‐CPQ	CPQ11‐14 (*η* ^2^)	P‐CPQ (*η* ^2^)
Definite	19 (16.2%)	36.47 ± 12.64	32.78 ± 14.35	14.25	14.21	< 0.001, (0.373)	< 0.001, (0.372)
Severe	15 (12.8%)	46.07 ± 21.68	45.53 ± 22.99				
Very severe	17 (14.5%)	74.29 ± 28.90	70.06 ± 25.45				

^a^ ANOVA, post hoc Tukey test: very severe > definite (*p* < 0.01), very severe > severe (*p* < 0.05), definite versus severe (*p* > 0.05). Higher CPQ11‐14 and P‐CPQ scores indicate poorer oral health‐related quality of life (OHRQoL).

Age‐stratified analyses showed that among children older than 12 years, CPQ11‐14 and P‐CPQ scores were significantly higher in those with malocclusion compared with DAI ≤ 25 group (*p* < 0.001 for both). In contrast, these differences were not statistically significant in children aged 12 years or younger (*p* = 0.066 for CPQ11‐14 and *p* = 0.130 for P‐CPQ). Furthermore, OHRQoL scores differed significantly across malocclusion severity categories in children older than 12 years (*p* = 0.001 for both questionnaires), whereas no significant differences were detected among severity groups in younger children (Table 3). Post hoc analyses in older children indicated that very severe malocclusion was associated with the highest OHRQoL scores and differed significantly from definite malocclusion, while other pairwise differences were less pronounced. These stratified findings should be interpreted cautiously due to limited subgroup sizes.

**Table 3:** Comparison of P‐CPQ and CPQ11‐14 scores between DAI severity groups, stratified by age group and by malocclusion severity within each age group.

Age				CPQ11‐14	P‐CPQ	Test statistics		*p* value (effect size)	
*n* (%)	Group	Malocclusion	Patient no.	(mean ± SD)	(mean ± SD)	CPQ11‐14	P‐CPQ	CPQ11‐14	P‐CPQ
≤ 12 75 (64.1%)	DAI ≥ 26	Definite	10	32.80 ± 14.37	26.40 ± 14.28	3.72	3.28	0.155^a^ (0.086^b^)	0.058^a^ (0.064^b^)
		Severe	8	39.25 ± 23.44	40.75 ± 20.93				
		Very severe	5	63 ± 33.82	57.40 ± 35.71				
		All	23	41.60 ± 24.67	38.13 ± 24.58	−1.87	−1.55	0.066^c^ (0.48^d^)	0.130^c^ (0.43^d^)
	DAI ≤ 25	Normal	37	31.43 ± 17.39	29.54 ± 12.29				
> 12 42 (35.9%)	DAI ≥ 26	Definite	9	40.55 ± 9.58	39.90 ± 11.23	9.46	9.50	0.001^e^ (0.43^f^)	0.001^e^ (0.43^f^)
		Severe	7	53.86 ± 17.97	51 ± 25.63				
		Very severe	12	79 ± 26.77	75.33 ± 19.36				
		All	28	60.36 ± 26.21	57.86 ± 24.32	−4.87	−4.58	< 0.001^g^ (1.55^d^)	< 0.001^g^ (1.48^d^)
	DAI ≤ 25	Normal	29	25.90 ± 17.60	26.03 ± 17.10				

*Note:* Higher CPQ11‐14 and P‐CPQ scores indicate poorer oral health‐related quality of life (OHRQoL).

“All” represents the combined DAI ≥ 26 group.

Abbreviations: DAI, Dental Aesthetic Index; DAI ≤ 25, normal occlusion or mild malocclusion; DAI ≥ 26, definite, severe, or very severe malocclusion.

^a^ Kruskal–Wallis test.

^b^ *ε* ^2^.

^c^ Independent samples *t*‐test.

^d^ Cohen's *d*.

^e^ ANOVA.

^f^ *η* ^2^.

^g^ Mann–Whitney *U* test.

Gender‐stratified analyses revealed that CPQ11‐14 scores were significantly higher in children with malocclusion than in the DAI ≤ 25 group in both girls (*p* < 0.001) and boys (*p* = 0.020). In addition, CPQ11‐14 scores differed significantly across malocclusion severity categories in both girls (*p* = 0.003) and boys (*p* < 0.001) (Table 4). Post hoc comparisons showed that, in both sexes, children with very severe malocclusion consistently exhibited the highest CPQ11‐14 scores and differed significantly from those with definite malocclusion, whereas other pairwise differences were smaller. Given the limited number of participants in some sex‐by‐severity subgroups, these findings are considered exploratory.

**Table 4:** Comparison of CPQ11‐14 scores between DAI severity groups, stratified by gender and by malocclusion severity within each gender group.

Gender				CPQ11‐14		
*n* (%)	Group	Malocclusion	Patient no	(mean ± SD)	Test statistics	*p* value (effect size)
Girls 75 (64.1%)	DAI ≥ 26	Definite	11	33.09 ± 9.87	7.13	0.003^a^ (0.32^b^)
		Severe	9	50.11 ± 21.97		
		Very severe	13	68 ± 29.54		
		All	33	51.48 ± 26.57	−4.76	< 0.001^c^ (1.16^d^)
	DAI ≤ 25	Normal	42	26.48 ± 16.01		
Boys 42 (35.9%)	DAI ≥ 26	Definite	8	41.12 ± 15.13	14.66	< 0.001^a^ (0.66^b^)
		Severe	6	40 ± 21.69		
		Very severe	4	94.75 ± 15.33		
		All	18	52.67 ± 28.46	−2.32	0.020^e^ (0.81^d^)
	DAI ≤ 25	Normal	24	33.42 ± 19.57		

*Note:* Higher CPQ11‐14 and P‐CPQ scores indicate poorer oral health‐related quality of life (OHRQoL).

Abbreviations: DAI, Dental Aesthetic Index; DAI ≤ 25, normal occlusion or mild malocclusion; DAI ≥ 26, definite, severe, or very severe malocclusion.

^a^ ANOVA.

^b^ *η* ^2^.

^c^ Independent samples *t*‐test.

^d^ Cohen's *d*.

^e^ Mann–Whitney *U* test.

In multivariable linear regression analyses adjusting for child age, sex, parental education, and family income, DAI score remained the only independent predictor of both child‐reported CPQ11‐14 scores (standardized *β* = 0.575, *p* < 0.001) and parent‐reported P‐CPQ scores (standardized *β* = 0.537, *p* < 0.001). No significant independent associations were observed for the demographic variables included in the models (Table 5).

**Table 5:** Multivariable linear regression analysis of factors associated with child‐ and parent‐reported OHRQoL scores.

	CPQ11‑14			P‑CPQ		
	*B* (SE)	*β*	*p* value	*B* (SE)	*β*	*p* value
Age of child (years)	2.07 (1.73)	0.095	0.233	2.66 (1.64)	0.131	0.108
Sex (boy vs. girl)	5.53 (3.92)	0.107	0.162	1.00 (3.73)	0.021	0.789
DAI score (continuous)	1.13 (0.15)	0.575	< 0.001	0.98 (0.15)	0.537	< 0.001
Family income	−1.31 (3.25)	−0.040	0.688	−0.40 (3.09)	−0.013	0.898
Parental education level	1.02 (3.31)	0.030	0.759	−1.51 (3.15)	−0.048	0.633

*Note:* Multivariable linear regression models were used to evaluate the independent association between malocclusion severity (DAI score) and oral health‐related quality of life outcomes. Models were adjusted for child age, child sex, parental education, and family income. Higher CPQ11‐14 and P‐CPQ scores indicate poorer OHRQoL.

## 4 Discussion

The present analytical cross‐sectional study evaluated the association between malocclusion severity, as determined by the DAI, and OHRQoL in children aged 11–14 years using both child‐ and parent‐reported measures. The findings demonstrated significant differences in OHRQoL scores across varying levels of malocclusion severity, as well as between children with malocclusion and those with normal occlusion.

The DAI is an orthodontic treatment index used to assess the orthodontic needs based on esthetic standards, and is sensitive to the social and emotional dimensions of the quality of life (Angkana et al. 2021). Because the consequences of malocclusion extend beyond clinical findings (Duisterwinkel et al. 2025), the use of validated instruments such as the CPQ11‐14 and P‐CPQ allows for a more comprehensive evaluation of its association with emotional, social, and functional dimensions of OHRQoL (Noronha et al. 2025; Heravi et al. 2011). Psychosocial factors such as self‐image and overall appearance play a key role in predicting the impact of malocclusion severity on the quality of life score (Duisterwinkel et al. 2025; Ramos et al. 2022).

The present findings showed that higher malocclusion severity was associated with progressively poorer OHRQoL scores, with the highest CPQ11‐14 and P‐CPQ scores observed in children with very severe malocclusion. This finding is in line with the results of Da da Rosa et al. (2016), Sun et al. (2020), J. K. Baskaradoss et al. (2022), and Nosratabadi et al. (2023) who showed that malocclusion negatively affects factors affecting the OHRQoL. This observed association may be partly explained by functional limitations in mastication and speech, as well as by esthetic concerns that can influence children's daily activities, self‐confidence, and social interactions (Koskela et al. 2021; Zakai et al. 2024). However, Locker et al. (2007), Dawoodbhoy et al. (2013), J. K. Baskaradoss et al. (2022), and Masoumi and Abdollahi (2020) did not observe a clear gradual trend for the mean quality of life score when comparing the three malocclusion groups, and the difference among the groups was not statistically significant. This discrepancy in the results may be due to differences in sample characteristics, and distribution of individuals and sample sizes among the four malocclusion categories (Heravi et al. 2011). In addition to statistical significance, the magnitude of the observed differences suggests clinical relevance. The mean differences in CPQ11‐14 and P‐CPQ scores between malocclusion groups exceeded 20 points, with large standardized effect sizes. Although a universally accepted minimal important difference for these instruments has not been firmly established, the magnitude of the observed score differences is substantial and likely reflects potentially clinically meaningful impacts on emotional, social, and functional domains of OHRQoL.

Sex‐based analyses showed that poorer OHRQoL was associated with malocclusion in both girls and boys, although statistically significant associations were observed in both sexes. This finding is in line with the findings of Sun et al. (2020), Ortiz et al. (2022), and Sfreddo et al. (2019). This difference could be due to the greater sensitivity of girls to the psychosocial consequences of malocclusion and a reflection of their esthetic concerns (Ortiz et al. 2022). However, J. K. Baskaradoss et al. (2022) and Tessarollo et al. (2012) found no significant difference in CPQ11‐14 total score between girls and boys, and showed that girls and boys were equally affected in all four CPQ11‐14 domains. A number of reasons may be responsible for lack of a significant difference in OHRQoL between girls and boys, such as differences in cultural characteristics and social attitudes that affect concerns and responses to esthetics and oral health in girls and boys in a similar way, which make these differences less visible in some communities (Rantavuori et al. 2023).

Age‐stratified analyses indicated statistically significant associations in children older than 12 years. In younger children, similar trends were observed; however, these did not reach statistical significance. This is consistent with the results of Anthony et al. (2018) and Kragt et al. (2016). This finding may be related to increased self‐awareness, social comparison, and concern with appearance during early adolescence (Jokovic et al. 2002). Nonetheless, previous findings on the relationship of age and OHRQoL have been inconsistent. Dimberg et al. (2016) reported that OHRQoL increased between the ages of 11 and 14 years, while Sun et al. (2020) showed that OHRQoL decreased between the ages of 12 and 14 years. Such inconsistent results may reflect the complexities of the adolescence period and the influence of multiple psychological, social, and cultural factors that influence the perception and reporting of OHRQoL (Göranson et al. 2023).

The P‐CPQ results in the current study showed that parents perceived the impact of malocclusion on their children's quality of life, although their scores were slightly lower than the children's self‐reports. This finding was in agreement with the results of studies by Oliveira et al. (2023) and Benson et al. (2010) which reported that parents and children generally agreed on the impact of malocclusion on the quality of life. However, Abreu et al. (2015) found a significant difference between adolescents and their parents' reports, with adolescents reporting more severe impacts of malocclusion. In a study by Çınar et al. (2025), there was a reasonable overall agreement between parents and children, but this agreement decreased in the emotional and social dimensions. This difference in views is likely due to psychological, cultural, and social differences, and indicates the importance of using both parent and child reports for a more comprehensive assessment of OHRQoL (Abreu et al. 2015). The age‐ and sex‐stratified analyses were exploratory in nature and should therefore be interpreted with caution.

Several limitations should be considered when interpreting the findings of this study. First, the use of convenience sampling from a single specialized dental clinic may limit the generalizability of the results and may have led to an overestimation of the association between malocclusion severity and OHRQoL. Children seeking care at a specialized orthodontic clinic may differ systematically from the general population in terms of treatment‐seeking behavior, subjective concern about dental appearance, functional complaints, and referral pathways. These characteristics may increase the likelihood of both more severe malocclusion and greater perceived impact on daily life, thereby potentially inflating observed differences in OHRQoL across malocclusion categories. Second, although multivariable linear regression models were applied to adjust for age, sex, parental education, and family income, residual confounding by unmeasured clinical or psychosocial factors cannot be completely excluded. Third, subgroup analyses stratified by age and sex were exploratory in nature and may have been underpowered due to small sample sizes within certain malocclusion severity categories; therefore, these findings should be interpreted with caution. Finally, despite excellent intra‑examiner reliability of DAI measurements following calibration, the use of a single examiner may still limit external reproducibility.

From a clinical perspective, the present findings emphasize that orthodontic assessment should extend beyond objective clinical indices. Considering the psychosocial dimensions associated with malocclusion severity may support more patient‐centered treatment planning and improve communication, satisfaction, and adherence to orthodontic care.

## 5 Conclusion

The findings of the present study indicate a significant association between malocclusion severity and OHRQoL in children aged 11–14 years. The magnitude of the observed differences suggests potentially clinically relevant impacts across emotional, social, and functional domains. Stratified analyses by age and sex showed differences in statistical significance across subgroups, which may partly reflect limited statistical power in smaller strata; therefore, these findings should be considered exploratory and interpreted with caution. Given the cross‐sectional design, causal inferences cannot be made. Although multivariable models were used to adjust for key demographic factors, residual confounding cannot be excluded. Overall, the results highlight the potential importance of considering both clinical and psychosocial dimensions when evaluating the broader impact of malocclusion. Further population‐based and longitudinal studies are needed to confirm these findings and clarify their clinical implications.

## Author Contributions

**Seyedeh Hediyeh Daneshvar:** conceptualization, methodology, writing – review and editing. **Negin Bazgir:** writing – original draft, data curation. **Sobhan Agheshteh Sabzevar:** resources, investigation, visualization. **Yasamin Babaee Hemmati:** funding acquisition, project administration, supervision.

## Funding

The authors received no specific funding for this work.

## Ethics Statement

This study was approved by the Ethics Committee of Guilan University of Medical Sciences, Rasht, Iran (Ethics code: IR.GUMS.REC.1400.616).

## Consent

Written informed consent was obtained from all parents or legal guardians of the participating children, and verbal assent was obtained from the children prior to data collection. All procedures were conducted in accordance with the ethical standards of the institutional and national research committees and with the 1964 Helsinki declaration and its later amendments.

## Conflicts of Interest

The authors declare no conflicts of interest.

## Data Availability

The datasets used and/or analyzed during the current study are available from the corresponding author on reasonable request. Also, the datasets supporting the conclusions of this article are included within the article.
